# The JAK2 GGCC (46/1) Haplotype in Myeloproliferative Neoplasms: Causal or Random?

**DOI:** 10.3390/ijms19041152

**Published:** 2018-04-11

**Authors:** Luisa Anelli, Antonella Zagaria, Giorgina Specchia, Francesco Albano

**Affiliations:** Department of Emergency and Organ Transplantation (D.E.T.O.), Hematology Section, University of Bari, 70124 Bari, Italy; luisa.anelli@uniba.it (L.A.); antonella.zagaria@uniba.it (A.Z.); giorgina.specchia@uniba.it (G.S.)

**Keywords:** *JAK2* germline haplotype, single nucleotide polymorphisms, myeloproliferative neoplasms

## Abstract

The germline *JAK2* haplotype known as “GGCC or 46/1 haplotype” (haplotype^GGCC_46/1^) consists of a combination of single nucleotide polymorphisms (SNPs) mapping in a region of about 250 kb, extending from the *JAK2* intron 10 to the Insulin-like 4 (*INLS4*) gene. Four main SNPs (rs3780367, rs10974944, rs12343867, and rs1159782) generating a “GGCC” combination are more frequently indicated to represent the *JAK2* haplotype. These SNPs are inherited together and are frequently associated with the onset of myeloproliferative neoplasms (MPN) positive for both *JAK2* V617 and exon 12 mutations. The association between the *JAK2* haplotype^GGCC_46/1^ and mutations in other genes, such as thrombopoietin receptor (*MPL*) and calreticulin (*CALR*), or the association with triple negative MPN, is still controversial. This review provides an overview of the frequency and the role of the *JAK2* haplotype^GGCC_46/1^ in the pathogenesis of different myeloid neoplasms and describes the hypothetical mechanisms at the basis of the association with *JAK2* gene mutations. Moreover, possible clinical implications are discussed, as different papers reported contrasting data about the correlation between the *JAK2* haplotype^GGCC_46/1^ and blood cell count, survival, or disease progression.

## 1. Introduction

Classic mutations of Janus Kinase 2 gene (*JAK2*), such as V617F in exon 14 and a large spectrum of mutations in exon 12, represent the molecular hallmark of polycythemia vera (PV), where they are found in more than 95% of patients [1,2,3,4]. The *JAK2* V617F mutation is also detected in approximately 50–60% of patients with primary myelofibrosis (PMF) or essential thrombocythemia (ET) [5]. To investigate the possible interplay between somatically acquired gene mutations and inherited genetic variations in patients with myeloproliferative neoplasms (MPNs), Pardanani et al. in 2008 studied the role of single nucleotide polymorphisms (SNPs) within four candidate genes involved in the *JAK-*Signal transducer and activator of transcription (*STAT*) signaling pathway, including receptors for erythropoietin (*EPOR*), thrombopoietin (*MPL*), granulocyte colony stimulating factor (*GCSFR*), and *JAK2* [6]. This study revealed for the first time a significant association between the occurrence of specific SNPs in *JAK2* gene and the onset of different MPNs [6]. Subsequently, in 2009, three independent groups revealed an interesting and strong association between the risk of developing a *JAK2* V617F positive MPN and a germline haplotype including the 3′ portion of *JAK2*, named GGCC or 46/1 haplotype (haplotype^GGCC_46/1^) [7,8,9]. The term haplotype stands for “haploid genotype” and refers to a group of genetic markers, generally represented by different SNPs, mapping on the same chromosome that are inherited together, as they are not separated by meiotic or mitotic recombination; having one of these genetic markers usually implies having all the others as well.

## 2. Genomic Architecture of the *JAK2* Haplotype^GGCC_46/1^

**Figure 1:**
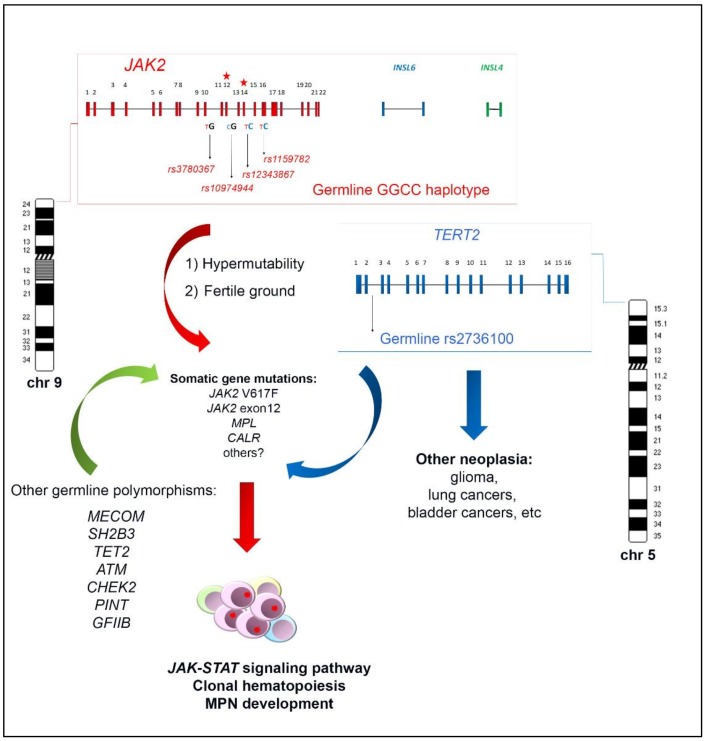
Schematic representation of the *JAK2* haplotype^GGCC_46/1^ genomic architecture and its possible interplay with the germline *TERT* rs2736100 in inducing somatic gene mutations, clonal expansion and MPN onset. Red, blu and green arrows refer to the possible induction of somatic gene mutations by the *JAK2* haplotype^GGCC_46/1^, *TERT* rs2736100, and other germline polymorphisms, respectively.

The haplotype^GGCC_46/1^ consists of hundreds of SNPs mapping in a region of about 250–280 Kb on chromosome 9p and including the three genes *JAK2*, Insulin-like 6 (*INSL6*) and Insulin-like 4 (*INSL4*) [8,10,11] (Figure 1). Among these genes, only *JAK2* is expressed in the hematopoietic tissue, whereas *INSL6* is preferentially expressed in the testis where it plays a role in sperm development and fertilization, and *INSL4* is highly expressed in the early placenta and influences trophoblast development and bone formation. The promoter region of *JAK2* gene is not included in the haplotype^GGCC_46/1^ as the genotyped SNPs in this region are not in linkage disequilibrium (LD) with the haplotype [8]. The *JAK2* 46/1 haplotype is also referred to as the “GGCC” haplotype, as can be represented by four main SNPs (rs3780367, rs10974944, rs12343867, and rs1159782) that replace three thymidines (T) and one cytosine (C) by two guanosines (G) and two cytosines, generating a “GGCC” combination (Figure 1) [8]. These four SNPs map on *JAK2* introns 10, 12, 14, and 15, respectively, and are always inherited together, being in complete linkage disequilibrium (Figure 1). As regards the origin of the “46/1 haplotype” definition, it derives from the study by Jones et al. that determined the haplotype structure of the *JAK2* gene using 14 SNPs genotyped by the Wellcome Trust Case Control Consortium (WTCCC) in 1500 healthy blood donors. Nine haplotypes were found to account for 94% of *JAK2* alleles and, among these, two haplotypes (numbers 46 and 1) were found to have a combined frequency of 0.24 in healthy individuals and to be identical except for one SNP [7]. These two haplotypes were found to be more frequently associated with *JAK2* V617F positive MPNs [7].

## 3. Frequency of the Haplotype^GGCC_46/1^ in MPN and Other Myeloid Neoplasms 

The frequency of the *JAK2* haplotype^GGCC_46/1^ in the healthy population is about 24%, whereas it was found in 40–80% of *JAK2* V617F positive MPN [7,8,12,13,14,15], in about 64% of cases bearing *JAK2* exon 12 mutations [10], and in approximately 36% of MPN cases bearing *MPL* mutations [16]. However, these last data were not confirmed by a subsequent study that found no association between the *JAK2* haplotype and *MPL* mutations [17]. The possible role of the *JAK2* haplotype^GGCC_46/1^ in *JAK2* V617F negative MPNs is still controversial, as some authors did not identify significant association in this group of patients [7,18]. On the other hand, other studies identified a weak association between the haplotype^GGCC_46/1^ and *JAK2* V617F negative MPN, suggesting that the presence of this germline condition confers a more generalized predisposition to MPN development, independently of the V617F mutation [7,12,16,19,20]. However, in 2010, calreticulin (*CALR*) gene mutations had not yet been identified, therefore the mutational status of V617F negative TE and MF patients could not be correctly assessed. After the identification of *CALR* mutations, further studies produced conflicting results regarding the frequency of the haplotype^GGCC_46/1^ in this group of MPN patients, mostly suggesting a lack of association [14,21,22,23]. Therefore, the possible association between the occurrence of the haplotype^GGCC_46/1^ and *JAK2* V617F negative MPN cases warrants further investigation. The presence of the haplotype^GGCC_46/1^ has also been investigated in chronic myeloid leukemia (CML) but no significantly increased frequency was revealed [24]. Analysis of the haplotype^GGCC_46/1^ in acute myeloid leukemia (AML) patients showed that the allele frequency did not significantly differ as compared to normal controls; however, an altered haplotype^GGCC_46/1^ frequency was observed in AML patients with and without normal karyotype (NK) (33–34% versus 12–25%) [13,25].

## 4. The Role of the *JAK2* Haplotype^GGCC_46/1^ and Other Germ Line Variants in Familial and Sporadic MPNs

As reviewed, the *JAK2* haplotype^GGCC_46/1^ is a germline inherited condition which confers a predisposition and an increased risk of developing MPN, preferentially but not exclusively in association with the *JAK2* V617F mutation (Figure 1). The identification of this germline variant raised the hypothesis that this inherited predisposition might explain familial MPN clustering. However, different studies investigated the haplotype^GGCC_46/1^ allele frequency in both familial and sporadic MPN in comparison with normal controls, without finding a significant difference in these two groups [26,27]. These findings suggest that that the *JAK2* haplotype^GGCC_46/1^ does not explain familial MPNs, which account for 5–10% of all MPN cases [28,29,30,31]. At the same time, it was found that the rs2736100 SNP, located in the second intron of the telomerase reverse transcriptase (*TERT*) gene, had a different allele frequency in familial MPN compared to sporadic cases [27] and exhibited a strong cancer predisposition effect in all MPN subtypes, regardless of the *JAK2* gene mutations occurrence [23,32]. The *TERT* gene at 5p15.33 encodes the catalytic subunit of the telomerase complex, playing an important role in maintaining telomere length [33]. Germline mutations in the *TERT* gene lead to dyskeratosis congenita, a disorder characterized by cancer susceptibility due to telomeres shortening [34]. The rs2736100_C allele of the *TERT* gene was previously shown to be associated with an elevated risk for several other neoplasia, such as glioma, lung and bladder cancers (Figure 1) [35,36,37,38]. A recent meta-analysis confirmed that the *TERT* rs2736100 polymorphism is associated with increased overall cancer risk, including solid cancers, myeloproliferative neoplasms, and acute myeloid leukemia [39]. The *JAK2* haplotype^GGCC_46/1^ and *TERT* rs2736100_C are independent factors predisposing to MPN and confering an additional disease risk (Figure 1) [22,27,40,41]. Moreover, it has been shown that MPN patients carrying *TERT* rs2736100_C have an increased risk of developing solid tumors, especially if treated with cytoreductive therapy [40]. A recent genome-wide association study was performed with a SNP array platform to identify novel predisposition alleles associated with the onset of MPNs and *JAK2* V617F clonal hematopoiesis in the general population [42]. This study confirmed the role of the *JAK2* haplotype^GGCC_46/1^ and *TERT* SNPs as germline factors predisposing to MPN but also identified significant associations between the occurrence of polymorphisms near *SH2B3*, *TET2*, *ATM*, *CHEK2*, *PINT*, and *GFI1B* genes and *JAK2* V617F clonal hematopoiesis and/or MPN development (Figure 1) [42]. Another recent study investigated the contribution of additional germline polymorphisms, such as *MECOM* rs2201862, *HBS1L-MYB* rs9376092 and *THRB-RARB* rs4858647, to the onset of MPN [23]. The SNP rs2201862, located downstream of *MECOM* gene, had the third strongest influence on the risk of developing MPN, after the *JAK2* 46/1 haplotype and *TERT* rs2736100 polymorphisms, *MECOM* rs2201862 was found to predispose especially to PV and to *CALR* mutated ET and PMF (Figure 1) [23].

## 5. Clinical Implications of the *JAK2*^46/1_GGCC^ Haplotype

The majority of studies failed to detect any association of the *JAK2* haplotype^GGCC_46/1^ with the distribution of age, sex or clinical parameters, including hemoglobin level, leukocyte count, rate of thrombosis/disease transformation and survival, suggesting that the *JAK2* haplotype^GGCC_46/1^ does not seem to affect the clinical phenotype or prognosis (Table 1) [12,19,20,43]. Preliminary data supporting a functional difference between alleles bearing or not the *JAK2* haplotype^GGCC_46/1^ were provided by Jones’ study in 2009, which investigated whether the haplotype^GGCC_46/1^ influences myeloid colony formation in healthy individuals showing that the presence of at least one 46/1 allele is associated with fewer circulating granulocyte-macrophage progenitor cells than are present without the 46/1 allele [7]. Another study showed that normal individuals bearing the *JAK2* haplotype^GGCC_46/1^ had slightly increased erythrocyte and decreased platelet count as compared to non-carriers [44]. However, these findings were not confirmed by further genome-wide association studies [45,46,47]. Tefferi et al., in 2010, found that MF patients negative for the *JAK2* haplotype^GGCC_46/1^ had a significantly shorter survival as compared to MF cases bearing the haplotype [12]; these data, however, were not confirmed by a subsequent analysis [20], whereas the study by Martínez-Trillos et al. in 2014 showed that MF patients with a homozygous 46/1 haplotype had significantly higher hemoglobin values and higher leukocyte counts but no association with other clinical characteristics [48]. As regards the prognostic significance of the JAK2 haplotype^GGCC_46/1^ in AML cases, it was demonstrated that cases with a normal karyotype bearing the haplotype^GGCC_46/1^ showed a trend towards myelomonocytic proliferation and shorter disease-free survival and overall survival compared to GGCC_46/1 non carriers; on the other hand, the haplotype^GGCC_46/1^ had no impact on prognosis in the subgroup of AML with an abnormal karyotype [13,25]. Moreover, interesting evidence reported an increased frequency of the *JAK2* haplotype^GGCC_46/1^ in patients with MPN characterized by splanchnic vein thrombosis (SVT), both in the presence and absence of the JAK2 V617F mutation [49,50,51,52,53]. A meta-analysis was performed on 26 observational studies involving 8561 cases, which showed that the *JAK2* haplotype^GGCC_46/1^ significantly raised the risk of development of MPNs and SVT [54]. Other evidence suggested a significant association between some SNPs included in the *JAK2* haplotype^GGCC_46/1^ (e.g., rs10758669) and inflammatory disorders such as ulcerative colitis and Crohn's disease [55,56,57]. In this regard, it is known that the *JAK2-STAT3* pathway is one of the most important cell signaling pathways in Crohn’s disease, being activated by the production of *IL-6* and is responsible for the production of pro-inflammatory proteins [58,59]. A possible explanation is that the haplotype^GGCC_46/1^ may cause an excessive production of cytokines with pro-inflammatory action which would further impair immune responses [11,60]. A recent study investigated the association of recipient and donor *JAK2* haplotype^GGCC_46/1^ and the outcome of allogeneic hematopoietic stem cell transplantation (allo-HSCT) in a series of 124 AML patients [60]. The findings from this study suggest that the occurrence of the *JAK2* haplotype^GGCC_46/1^ in both recipients and donors significantly affected the development of acute graft-versus-host disease, confirming that *JAK2* polymorphisms may have an influence on cytokines signaling pathways [61]. 

## 6. Correlation between the *JAK2* Haplotype^GGCC_46/1^ and *JAK2* V617F Allele Burden

Several reports showed that the haplotype^GGCC_46/1^ seems to be associated with a high mutant allele burden in *JAK2* V617F positive MPN patients, being significantly enriched in patients with higher V617F allele burden [12,18,19,20,21]. These data suggest that the *JAK2* haplotype^GGCC_46/1^ could confer a possible selective advantage to the V617F mutant clone as well as promoting the acquisition of *JAK2* V617F mutation. Moreover, the *JAK2* haplotype^GGCC_46/1^ might have an influence on the occurrence of the 9p mitotic recombination, causing homozygosity of the *JAK2* V617F mutation [18]. Alvarez-Larrán et al. investigated the influence of the *JAK2* haplotype^GGCC_46/1^ during disease follow-up revealing that PV patients homozygous for the haplotype show a progressive and higher increase in the *JAK2* V617F allele burden during the disease evolution without cytoreductive therapy, as compared to patients with a negative or heterozygous haplotype [43]. However, a recent report found no significant difference in the *JAK2* haplotype^GGCC_46/1^ frequency between groups of MPN patients with different *JAK2* V617F allele burdens [15], therefore this aspect remains to be further clarified.

## 7. Potential Mechanisms Explaining the Association between the *JAK2* Haplotype^GGCC_46/1^ and *JAK2* V617F Mutation

There are two principal hypotheses that aim to explain the association between the germline haplotype^GGCC_46/1^ and the *JAK2* V617F somatic mutation (Figure 1). The “hypermutability hypothesis” derives from the observation that MPN patients heterozygous for the haplotype^GGCC_46/1^ preferentially acquire the V617F mutation in cis with the GGCC predisposition allele [7,8,9]; this germline haplotype may somehow determine an increase in the mutation rate at the *JAK2* locus and those mutations that confer a selective advantage, such as *JAK2* V617F, would cause a clonal myeloproliferative disorder [9,62]. *JAK2* exon 12 mutations are also preferentially acquired in cis with this haplotype, supporting the hypermutability hypothesis. It is possible that a “regulatory environment” of an unknown nature is present on the haplotype^GGCC_46/1^ and renders DNA more susceptible to damage or replication errors; this could promote the acquisition of gene mutations in cis and induce clonal expansion and the onset of MPNs. It is also plausible that this cis regulatory environment could promote alterations of gene expression, although the haplotype^GGCC_46/1^ does not include the promoter region of the *JAK2* gene. However, no genotype-specific differences in *JAK2* gene expression were observed [7,9,63]. Moreover, it was shown that the SNPs within the haplotype^GGCC_46/1^ are not in LD with nonsynonymous SNPs that might alter protein function and structure [8]. It cannot be excluded that the haplotype^GGCC_46/1^ is associated with an altered expression of *INSL6* or *INSL4* genes, which are not normally expressed in hematopoietic cells; their abnormal activation could eventually lead to an altered cytokine production, concurring in stimulation of inflammation pathways [11], but further studies are needed to clarify this matter. The second possible explanation, named the “fertile ground hypothesis”, suggests that *JAK2* mutations arise on all haplotypes at the same rate, but the GGCC_46/1 allele confers a selective advantage to the *JAK2* V617F positive clone. In this hypothesis, the haplotype^GGCC_46/1^ could provide a global DNA propensity for gene mutations and MPN development, as some conflicting evidence indicates that this haplotype could also be associated to *MPL* or *CALR* gene mutations [16,17,22,62]. Moreover, this second hypothesis could explain the unclear observations that the haplotype may also be enriched in individuals with *JAK2* V617F negative MPNs [7,12,13,19,20,39]. The “fertile ground hypothesis” could also explain the acquisition of mutations in other genes that are critical for expansion and differentiation of myeloid cells, possibly causing malignant transformation to MPN or AML [13].

## 8. Conclusions

About ten years after its discovery, the possible pathogenic role of the JAK2 haplotype^GGCC_46/1^ in MPN patients, as well as in other myeloid malignancies, is not yet understood. The association with JAK2 mutations has been largely confirmed, whereas there is no agreement about the frequency in JAK2 V617F negative or MPL and CALR mutated MPN patients. The most probable explanation is that the JAK2 haplotype^GGCC_46/1^ could influence the activation of the constitutive JAK-STAT signaling pathway rather than a specific mutation, supporting clonal hematopoietic proliferation. The mechanism of this possible activation, however, needs further investigations.

## Figures and Tables

**Table 1:** Main literature studies describing the frequency of the JAK2 haplotype^GGCC_46/1^ in myeloid malignancies and possible clinical correlations.

Reference	Main Findings	Association between the GGCC Haplotype and Somatic Mutations	Association between the GGCC Haplotype and *JAK2* V617F Neg MPN	Tagging SNPs	Series of Analyzed Patients	Clinical Findings
[6]	First identification of *JAK2* SNPs significantly associated with PV or ET	-	-	rs7046736, rs10815148, rs12342421	84 PV, 58 PMF, and 37 ET	Association between *JAK2* SNPs and leucocytosis, higher hemoglobin level, lower platelet count
[7]	Association between *JAK2* 46/1 haplotype and *JAK2* V617F positive MPN	*JAK2* V617F (48–56%)	Weak association	rs12340895	88 PMF	Hematologically normal individuals that carried at least one 46/1 allele grew significantly fewer CFU-GM
[19]	The incidence of the 46/1-linked C allele was significantly higher in ET than in population controls	*JAK2* V617F (44%)	Significant association	rs12343867	226 ET	The clinical features of 46/1 positive and negative ET were indistinguishable, including blood counts, rate of thrombosis/disease transformation and survival
[12]	JAK2 germline genetic variation affects disease susceptibility in PMF regardless of VF mutational status	*JAK2* V617F (50%)	Significant association	rs12343867	130 MF	Association between nullizygosity for the JAK2 46/1 haplotype SNP allele and shortened survival
[55]	The SNP rs10758669_C allele increase the risk of having Crohn’s disease	-	-	rs10758669	302 Crohn’s disease	This JAK2 variant strongly enhanced the risk of ileocolonic disease, with stricturing or ileal/stricturing behavior, requiring a bowel resection
[16]	The frequency of 46/1 was higher in *MPL* mutated cases compared with controls	*MPL* W515K/L mutations (36%)	The 46/1 haplotype was also overrepresented in cases without V617F mutation	rs12340895	176 *MPL* pos/V617F neg ET, and 212 V617F pos ET	No association between 46/1 and clinical or laboratory features
[20]	The frequency of the 46/1 haplotype, was significantly higher in PMF patients showing the highest V617F allele burden	*JAK2* V617F (38.6%)	No statistical significant association	rs12343867	202 PMF	No statistically significant correlations between any of the possible rs12343867 genotypes and hematological or clinical variables
[13]	The 46/1 haplotype is a predisposition factor for JAK2 V617F positive MPN, and is also significantly associated with AML patients with normal karyotype	*JAK2* V617F (85%)	Significant association	rs12343867	312 MPN, 339 AML	The 46/1 haplotype is not associated with MPN manifestations, like disease type, splenomegaly, signs of increased erythropoiesis or myelopoiesis and vascular complication, except the increased risk of the development of myelofibrosis in homozygous cases
[50]	The 46/1 haplotype was overrepresented in *JAK2*V617F positive SVT patients compared with controls	*JAK2* V617F (43%)	*JAK2*V617F negative SVT patients with a proven MPN also exhibited an increased frequency of the 46/1 haplotype	rs12343867	199 SVT	The 46/1 haplotype was associated with increased erythropoiesis (higher hemoglobin levels, hematocrit, and red blood cell count) in *JAK2* V617F negative SVT patients
[25]	Association of the *JAK2* 46/1 haplotype with disease characteristics and treatment outcome in AML patients	-	-	rs12343867	176 AML	The 46/1 haplotype was found to be a factor predisposing to the development of acute myelomonocytic leukemia. In NK-AML, the carriers of 46/1 haplotype are characterized by shorter disease-free survival and overall survival
[43]	Untreated PV patients with homozygous JAK2 46/1 haplotype experienced a progressive increase in the *JAK2* V617F allele burden	*JAK2* V617F (68%)	-	rs12340895, rs12343867	26 PV, 36 ET	The 46/1 JAK2 haplotype status was not statistically different according to age, gender, type of diagnosis (PV or ET) or baseline hematological values
[48]	Among *JAK2* V617F positive patients, those who were homozygous for the 46/1 haplotype had a higher allele burden	*JAK2* V617F (40%)	-	rs12340895	132 MF	Patients with homozygous 46/1 haplotype showed significantly higher hemoglobin values and leukocyte counts, but no association was seen with other clinic hematologic features
[21]	The frequency of 46/1 haplotype was significantly higher in *JAK2* V617F positive PV/ET but not in ET patients with *CALR* mutations	*JAK2* V617F (40–50%)	No statistical significant association	rs12340895	72 PV, 115 ET	The presence of 46/1 haplotype had a trend to have higher white blood cell count in *JAK2* V617F mutated PV and ET patients but not in *CALR* mutated ET
[61]	Both, recipient and donor 46/1 haplotypes significantly affected aGvHD grades II–IV development	-	-	rs12343867	124 AML	The recipient haplotype remained independently related to aGvHD, while the donor not. Significantly less relapses were observed among haplotype carriers, but overall survival did not differ

## Abbreviations

PV	polycythemia vera
PMF	primary myelofibrosis
ET	essential thrombocythemia
MPN	myeloproliferative neoplasms
SNP	single nucleotide polymorphisms
EPOR	receptor for erythropoietin
MPL	receptors for thrombopoietin
GCSFR	colony stimulating factor
INSL4	insulin-like 4
INSL6	insulin-like 6
WTCCC	Wellcome Trust Case Control Consortium
CALR	calreticulin
CML	chronic myeloid leukemia
AML	acute myeloid leukemia
NK	normal karyotype
TERT	telomerase reverse transcriptase
allo-HSCT	allogeneic hematopoietic stem cell transplantation
